# Genome sequence of *Mycobacterium smegmatis* phage Mao1

**DOI:** 10.1128/mra.01080-24

**Published:** 2025-10-24

**Authors:** Mackenzie L. Kluttz, Catherine G. Griffin, Nathan A. Simpson, Lydia M. Suplita, Alison E. Kanak

**Affiliations:** 1 Biology Department, University of North Georgia 32193, Dahlonega, Georgia, USA; Portland State University, Portland, Orlando, USA

**Keywords:** bacteriophages, genomes

## Abstract

Mao1, a temperate bacteriophage isolated in North Georgia, was found to have a 65,240 base pair genome with 101 confirmed genes, no tRNA genes, and a 66.3% guanine–cytosine content. It shares 99.79% nucleotide identity with phage Sejanus. Bacteriophages that share over 50% identities are grouped into clusters, with Mao1 being in cluster AD.

## ANNOUNCEMENT

Bacteriophages can potentially fight antibiotic drug resistance by using phage therapy (1). To do this, phage development and diversity must be researched. This aims to contribute to the knowledge of phage by presenting bacteriophage Mao1, a temperate phage in cluster AD that infects *Mycobacteria smegmatis* mc^2^155.

**Fig 1:**
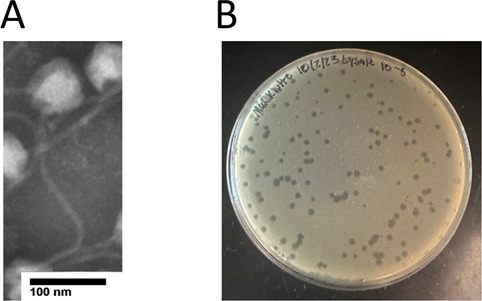
(**A**) Transmission electron micrograph (TEM) of Mao1. The TEM was obtained using a JEM-1011 TEM (JEOL, Inc., Tokyo, Japan) at the University of Georgia Electron Microscope Laboratory. (**B**) A plated sample of Mao1 at a dilution factor of 10^−5^ was incubated at 37°C for 48 h.

Mao1 was isolated from enriched soil at the University of North Georgia in Dahlonega, Georgia (34.527776 N, 83.986272 E) in dry, sandy soil. The phage was isolated using protocols provided by the Science Education Alliance-Phage Hunting Advancing Genomics and Science (2). Briefly, 7H10 liquid medium was added to the soil sample, incubated at 37°C for 24 h, and filtered using a 0.22 µm filter. This filtrate was incubated with *Mycobacteria smegmatis* mc^2^155 at 37°C and refiltered. Once plaques were detected via traditional plaque assay, three rounds of purification were performed. The titer was amplified to extract the genomic DNA for sequencing. Electron microscopy using phosphotungstic acid stain imaged Mao1. Mao1 was identified as having a siphovirus morphology with a capsid and tail measuring approximately 67.603 and 278.974 nm, respectively (Fig. 1A). Mao1’s plaques are approximately 3 mm with distinct, circular edges (3) (Fig. 1B).

The phage genomic DNA was isolated from lysate and created with a web plate using the Wizard DNA Extraction Kit (Promega) per manufacturer instructions. An NEB Ultra II Library Kit 9 with v3 Reagents and 150-base reads was used to synthesize a library. Mao1 was found to consist of 66.3% guanine–cytosine and 65,240 base pairs long and circularly permuted ends and was assigned with cluster AD. There were 325,500 single-end reads from the library. These raw reads were assembled using Newbler v2.9 with default settings. The resulting single-phage contig was checked for completeness, accuracy, and phage genomic termini using Consed v29 (4).

Mao1’s genome was annotated using Glimmer v3.02 (5), GeneMark v2.5p (6), DNA Master v4.2.1.11, Phamerator v557 (7), Starterator v557, NCBI BLASTp v2.15.0 (8), HHPred v2.08 (9), TMHMM v.1.0.24 (9), and PECAAN v20240320 (10). Software used was run with default parameters. Hits with E values of 10^e−10^ or less were considered acceptable. Phamerator and GeneMark indicated that Mao1 has 101 open reading frames (ORFs) with the ability to assign function to 36. Genes 1–38, 49–90, 93–95, and 97–99 read in the forward direction, while genes 39–48, 91–92, 96, and 100–101 read in the reverse direction. Mao1 is predicted to be temperate, as a tyrosine integrase (ORF43) was identified. Mao1 is mostly like Sejanus (GenBank accession no. OP172873), with 99.79% nucleotide identity via BLAST nucleotide alignment. Of interest, there is one base pair insertion found within ORF3, causing a frameshift resulting in a single long ORF rather than the two observed in other cluster members. The latter half of Mao1’s ORF 3 aligns 1:1 with Sejanus’ ORF 4 and the front half aligns almost 1:1 with Sejanus ORF 3 via BLASTp. Mao1’s genome has synteny with its cluster members in the front half of the genome, while the latter half loses most of its synteny, demonstrating mosaicism commonly found in phage genomes (11).

## Data Availability

Mao1 is available at GenBank with accession no. PP978845 and Sequence Read Archive (SRA) no. SRX25999123.

## References

[1] Petrovic Fabijan A, Iredell J, Danis-Wlodarczyk K, Kebriaei R, Abedon ST. 2023. Translating phage therapy into the clinic: recent accomplishments but continuing challenges. PLoS Biol 21:e3002119. doi:10.1371/journal.pbio.3002119 37220114 PMC10204993

[2] Poxleitner M, Pope W, Jacobs-Sera D, Sivanathan V, Hatfull GF. 2018. HHMI SEA-PHAGES phage discovery guide. Available from: https://seaphagesphagediscoveryguide.helpdocsonline.com/home

[3] Kluttz M. 2023. Mycobacterium phage Mao1. The actinobacteriophage database. Available from: https://phagesdb.org/phages/Mao1

[4] Russell DA. 2018. Sequencing, assembling, and finishing complete bacteriophage genomes, p 109–125. In Clokie MRJ, Kropinski AM, Lavigne R (ed), Bacteriophages: methods and protocols. Springer, New York, NY. 10.1007/978-1-4939-7343-9_9 29134591

[5] Delcher AL, Bratke KA, Powers EC, Salzberg SL. 2007. Identifying bacterial genes and endosymbiont DNA with Glimmer. Bioinformatics 23:673–679. doi:10.1093/bioinformatics/btm009 17237039 PMC2387122

[6] Besemer J, Borodovsky M. 2005. GeneMark: web software for gene finding in prokaryotes, eukaryotes and viruses. Nucleic Acids Res 33:W451–4. doi:10.1093/nar/gki487 15980510 PMC1160247

[7] Cresawn SG, Bogel M, Day N, Jacobs-Sera D, Hendrix RW, Hatfull GF. 2011. Phamerator: a bioinformatic tool for comparative bacteriophage genomics. BMC Bioinformatics 12:2105–12 doi:10.1186/1471-2105-12-395 PMC3233612 21991981

[8] Altschul SF, Gish W, Miller W, Myers EW, Lipman DJ. 1990. Basic local alignment search tool. J Mol Biol 215:403–410. doi:10.1016/S0022-2836(05)80360-2 2231712

[9] Söding J, Biegert A, Lupas AN. 2005. The HHpred interactive server for protein homology detection and structure prediction. Nucleic Acids Res 33:W244–8. doi:10.1093/nar/gki408doi:15980461 PMC1160169

[10] Hallgren J, Tsirigos KD, Pedersen MD, Almagro Armenteros JJ, Marcatili P, Nielsen H, Krogh A, Winther O. 2022. DeepTMHMM predicts alpha and beta transmembrane proteins using deep neural networks. BioRxiv. doi:10.1101/2022.04.08.487609

[11] Hatfull GF, Hendrix RW. 2011. Bacteriophages and their genomes. Curr Opin Virol 1:298–303. doi:10.1016/j.coviro.2011.06.009 22034588 PMC3199584

